# Ultrasound-guided *versus* neurostimulation-guided bilateral transperineal pudendal nerve block for analgesia in outpatient haemorrhoid surgery: protocol for a multicentre, randomised, double-blind, non-inferiority trial

**DOI:** 10.1016/j.bjao.2025.100512

**Published:** 2025-12-09

**Authors:** Thomas Giral, Olivier Maupain, Yoann Elmaleh, Davy Huynh, Floriane Ciceron, Kilian Yao, Pablo Devidas, Albert El Metni, Johannes Kutter

**Affiliations:** 1 Department of Anaesthesia, Hôpital Privé Claude Galien, Quincy-sous-Sénart, France; 2 Department of Anaesthesia, Clinique des Côtes du Rhône, Roussillon, France; 3 Department of Anaesthesia, Groupe Hospitalier Diaconesses Croix Saint-Simon, Paris, France

**Keywords:** haemorrhoid surgery, neurostimulation, non-inferiority trial, postoperative analgesia, pudendal nerve block, randomised controlled trial, regional anaesthesia, ultrasound

## Abstract

**Background:**

Haemorrhoid surgery is often associated with severe postoperative pain. Bilateral transperineal pudendal nerve block, performed using anatomical landmarks or nerve stimulation, is effective and currently recommended for postoperative analgesia. However, these blind techniques carry risks such as pudendal artery puncture or rectal injury and are not always routinely performed. The ultrasound-guided transperineal pudendal nerve block has shown promise in paediatric urological surgery, appearing both effective and well tolerated, but has not yet been evaluated in proctological procedures.

**Methods:**

This multicentre, prospective, randomised, double-blind trial aims to compare the analgesic efficacy, adverse effects, and safety of ultrasound-guided *vs* nerve stimulator-guided bilateral transperineal pudendal nerve block in patients undergoing haemorrhoid surgery. The primary endpoint is the maximum pain score (0–10 numerical rating scale) in the post-anaesthesia care unit. Secondary outcomes include length of stay in post-anaesthesia care unit and hospital, quality of recovery score (QoR-15 score), pain and analgesic medication consumption during the first 7 days, and treatment-related adverse effects and safety. A total of 202 patients are required to test non-inferiority, based on a predefined margin of 0.7. The study received ethical approval from the Comité de Protection des Personnes Ouest IV in May 2025.

**Conclusions:**

Recruitment is expected to begin in September 2025. Results will be submitted for peer-reviewed publication and conference presentation.

**Clinical trial registration:**

NCT07015775.

Haemorrhoidectomy is associated with significant postoperative pain, with mean visual analogue scale (VAS) scores ranging from 5 to 6/10 in the immediate postoperative period, often worsening during the first bowel movements.^1^^,^^2^ Effective pain management is therefore essential. Current guidelines^3^^,^^4^ recommend a multimodal analgesic strategy comprising regular administration of a non-steroidal anti-inflammatory drug (NSAID), such as ketoprofen, combined with paracetamol, and rescue opioid analgesia for pain exceeding 4/10. In addition, intraoperative injection of a long-acting local anaesthetic into the ischiorectal fossa—adjacent to the pudendal nerve and its branches—is a key component. This technique, commonly referred to as a pudendal nerve block, has been shown to reduce postoperative pain, decrease opioid consumption, and minimise opioid-related side-effects such as nausea, vomiting, constipation, and delayed gastrointestinal recovery.^5^, ^6^, ^7^

The pudendal nerve, composed of motor, sensory, and autonomic fibres, arises from the sacral roots (S2–S4), exits the pelvis via the greater sciatic notch, loops around the ischial spine, and enters Alcock’s canal after passing between the sacrospinous and sacrotuberous ligaments. It gives rise to several terminal branches innervating the perineum. Sensory blockade of the pudendal nerve can be achieved via two main approaches. The transgluteal route is associated with a high risk of transient sciatic nerve involvement (10–20%),^8^, ^9^, ^10^, ^11^ making it unsuitable for outpatient procedures such as haemorrhoid surgery. The second approach is transperineal, involving injection of local anaesthetic into the ischiorectal fossa, located between the ischium and the anal canal, below the levator ani and coccygeus muscles. This method is currently recommended for postoperative analgesia after haemorrhoid surgery.^3^^,^^4^ It is typically performed using neurostimulation to elicit sphincter contraction and confirm nerve proximity. However, this ‘blind’ technique carries a risk of pudendal artery puncture and possible rectal injury, limiting its routine use. Additionally, obtaining an appropriate motor response with the nerve stimulator can sometimes be challenging. In a large multicentre German study of 770 open haemorrhoidectomies, only 13.5% of patients received a pudendal nerve block.^1^ An ultrasound-guided transperineal pudendal nerve block has been described in paediatric urological surgery,^12^^,^^13^ demonstrating excellent analgesic efficacy and safety, with no sciatic nerve involvement. This technique is used in routine practice by the anaesthesia team at Hôpital Privé Claude Galien. To our knowledge, this is the first randomised controlled trial evaluating the ultrasound-guided transperineal pudendal nerve block for postoperative analgesia in adult haemorrhoid surgery.

The primary objective of this study is to test the non-inferiority of the ultrasound-guided transperineal pudendal nerve block compared with the standard neurostimulation-guided technique for postoperative analgesia in terms of postoperative pain, evaluated by maximal numerical rating scale (NRS) in the post-anaesthesia care unit (PACU). Secondary objectives include comparison of opioid requirements, procedure duration, pain evolution and functional recovery between both techniques. Although adverse events and safety outcomes will be monitored and reported, the study is not powered to detect significant differences in their incidence. This trial aims to generate robust comparative data and evaluate whether the ultrasound-guided technique can safely replace the neurostimulation-guided approach as standard care in proctological surgery.

## Methods

### Patient and public involvement, trial design

**Fig 1:**
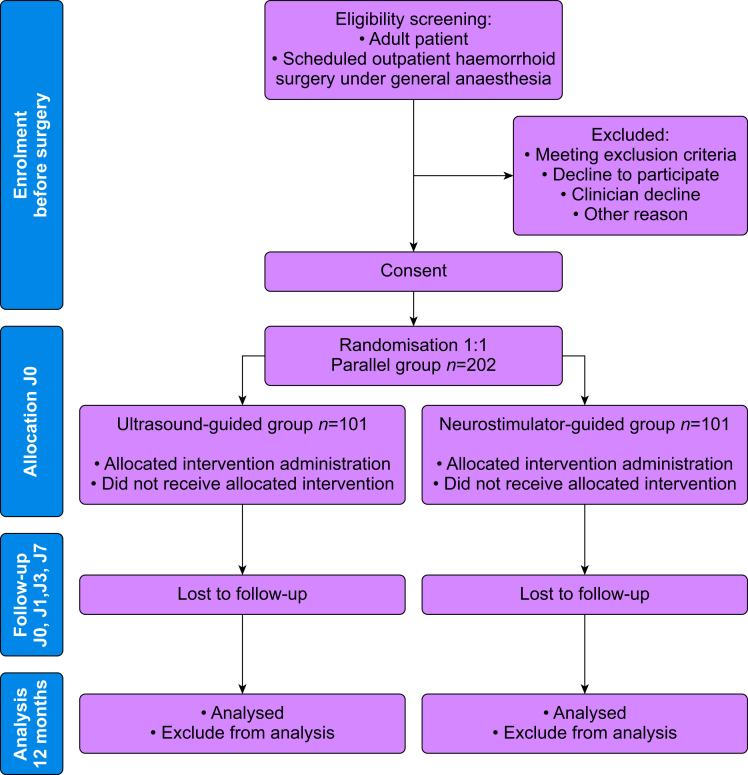
Study design.

A limitation of this study is the absence of a formal Patient and Public Involvement (PPI) process; however, informal feedback from patients regarding their postoperative experience and from members of the public on the clarity of information sheets was taken into account. This is a multicentre, prospective, randomised, double-blind, non-inferiority clinical trial with two parallel groups. Participants will be randomly assigned to receive a bilateral pudendal nerve block either guided by neurostimulation or by ultrasound. This study follows SPIRIT 2025 Checklist. A SPIRIT schedule is provided in Supplementary Materials. A graphical summary of study activities is shown in Figure 1.

### Participants, interventions, and outcomes

This trial will be conducted in three private hospitals in France: Hôpital Privé Claude Galien (Quincy-sous-Sénart), Clinique des Côtes du Rhône (Roussillon), and Groupe Hospitalier Diaconesses Croix Saint-Simon (Paris). All centres are equipped with ambulatory surgical facilities and PACUs. The trial will take place in operating theatres and postoperative recovery areas. Participants will be recruited from patients scheduled for outpatient haemorrhoid surgery. Eligibility screening will be performed during preoperative anaesthesia consultations. Study information sheets and oral explanations will be provided at this time, and written informed consent will be obtained before surgery. Randomisation will occur on the day of surgery, and participants will receive either an ultrasound-guided or neurostimulation-guided bilateral pudendal nerve block before the procedure. Recruitment is expected to be completed within 12 months.

Inclusion criteria are adult patients (≥18 yr) undergoing outpatient haemorrhoid surgery under general anaesthesia who provide written informed consent (including pedicular haemorrhoidectomy, stapled haemorrhoidopexy, or haemorrhoidal artery ligation with mucopexy). Exclusion criteria include prior haemorrhoid surgery, contraindication to study medications, spinal anaesthesia, contraindication to the use of a supraglottic airway device, chronic pain requiring opioids, cognitive or language impairments, lack of affiliation with the French health system, legal protection status, pregnancy or inadequate contraception, and participation in another clinical trial.

All procedures will be performed under general anaesthesia using a supraglottic airway device. Anaesthesia will be induced with i.v. propofol (2–4 mg kg^−1^) and sufentanil (0.2 μg kg^−1^), and maintained with sevoflurane (1–3%). Metronidazole (1 g), dexamethasone (8 mg), and droperidol (0.625–1.25 mg) will be administered unless contraindicated. The pudendal nerve block will be performed under general anaesthesia with the patient in the lithotomy position. Patients will be randomly assigned (1:1) to the neurostimulation-guided or ultrasound-guided group.

• In the neurostimulation group, after antiseptic preparation, a 22 G, 50 or 80 mm insulated needle (Stimuplex Ultra 360, BBraun, Melsungen, Germany) will be inserted at the midpoint between the anus and the ischial tuberosity, connected to a stimulator (Stimuplex HNS12, BBraun). Electrical stimulation will begin at 2.5 mA with a frequency of 1 Hz. The needle will be advanced to a depth of 4–5 cm until a contraction of the anal sphincter is observed. Once a response is obtained, the current will be reduced to 0.5 mA to confirm appropriate needle placement. If no response is observed, current intensity will be temporarily increased. A strong response at <0.3 mA will prompt repositioning to avoid intraneural injection. After negative aspiration, ropivacaine 15 ml (3.5 mg ml^−1^) will be injected incrementally. The procedure will be repeated contralaterally. If no response is obtained, the block will be performed blindly at a depth of 4 cm.
• In the ultrasound group, after antiseptic preparation, a 6 MHz convex probe will be placed transversely on the perineum, between the anus and the ischial tuberosity. The pudendal artery will be identified near the ischial tuberosity. A 22 G, 50 or 80 mm echogenic needle (Ultraplex 360, BBraun, Melsungen, Germany) will be inserted out-of-plane in the area on the medial side of the artery. After negative aspiration, ropivacaine 15 ml (3.5 mg ml^−1^) will be injected slowly. The spread is expected within the ischiorectal fossa. The procedure will then be repeated on the contralateral side.

Block fidelity will be assessed by visualisation of local anaesthetic spread adjacent to the pudendal artery in the ultrasound group, and by the minimal stimulation current eliciting anal sphincter contraction in the neurostimulation group. All investigators were trained and had each performed at least 10 procedures with each technique before trial initiation.

Postoperative analgesic management is standardised, with systematic administration of paracetamol, ketoprofen, and nefopam, and the use of opioid analgesics if pain control is insufficient.

The primary outcome is the maximum pain score in the PACU before opioid administration, assessed using a 0–10 NRS. The NRS was chosen as it is the standardised measure of pain intensity routinely used in the PACUs of all participating centres. Given the variability in length of stay in ambulatory haemorrhoid surgery, more complex or delayed assessments (e.g. repeated measures, early quality of recovery score [QoR-15] ) would carry a higher risk of missing data. The NRS thus provides a simple, robust, and homogenous endpoint across centres. Secondary outcomes include opioid consumption in PACU (converted to i.v. morphine equivalents), nerve block duration, block procedure time, pain scores at rest and during movement at 24 h, 3 and 7 days, total analgesic use over 7 days, timing and pain intensity of first defecation, PACU and hospital stay durations, and adverse events (e.g. sciatic nerve block, haematoma, vascular puncture, rectal injury). Functional recovery will be evaluated with the QoR-15 score on postoperative days 1, 3, and 5.

All adverse events will be documented from randomisation until postoperative day 7 through direct questioning, medical record review, and nursing reports. Each event will be graded (mild, moderate, severe) and assessed for causality (unrelated, possible, probable, definite). Serious adverse events will be reported to the sponsor within 24 h in accordance with French regulations. Based on institutional data (mean pain score 3.4 [2.0]), a non-inferiority margin of 0.7, standard deviation of 2.0, 80% power and one-sided α=0.05, a sample size of 101 patients per group is required. Thus, 202 patients will be enrolled.

### Assignment of interventions

The random allocation sequence will be generated by an independent biostatistician using computer-generated random numbers with randomly permuted block sizes to ensure group balance. No stratification will be applied. Randomisation will be implemented via the electronic case report form (eCRF). Once a participant is enrolled and consented, the investigator enters their data into the eCRF, which automatically assigns them to one of the two intervention groups based on the concealed allocation sequence. The assigned group is then communicated to the designated anaesthesiologist by automated e-mail. This system ensures allocation concealment, as investigators responsible for enrolment and outcome assessment remain blinded to the sequence and block sizes. The allocation sequence is securely stored and inaccessible to trial personnel. Participants and outcome assessors will remain blinded throughout the trial. Only the anaesthesiologist performing the block will be informed of group allocation, and this person must not be involved in enrolment or postoperative outcome assessment (except for block-related procedural data). Unblinding will be permitted only when necessary for immediate clinical management or participant safety. In such cases, the principal investigator may contact the randomisation manager to access the allocation. All unblinding events will be documented, including the reason and requester’s identity.

### Data collection, management, and analysis

Clinical data will be collected using a secure eCRF developed with ENNOV Clinical® (ENNOV, Paris, France) and hosted on general data protection regulation (GDPR)-compliant servers located in France. Investigators will receive secure credentials and enter data directly into the eCRF. Paper CRFs may be used as backup and transcribed subsequently.

Subjective assessments (e.g. pain scores, QoR-15) will be conducted by the same assessor when possible. All entries, corrections, and validations will be made by investigators or authorised site staff. The eCRF includes automated range and consistency checks to enhance data quality. The study will be monitored by the sponsor or its representative through on-site visits to ensure concordance between source data and CRFs. Data queries will be issued and resolved accordingly. Final quality checks will be conducted in accordance with the statistical analysis plan. Essential documents will be archived for a minimum of 15 yr, as required by French regulations. Data access will be strictly controlled and confidential. Audits or inspections will be allowed in compliance with these standards.

Based on preliminary data (mean NRS 3.4 [2.0]), a total of 202 patients (101 per group) is required to test non-inferiority, standard deviation of 2.0, one-sided alpha of 0.05, and 80% power. The non-inferiority margin was set at 0.7 NRS points (20% of the expected mean score), as smaller differences were not clinically relevant and larger ones were considered unacceptable. As the trial follows the intention-to-treat principle, crossovers will be analysed; if patients withdraw consent before surgery, replacements will be recruited to reach 202 participants. A detailed statistical analysis plan will be finalised before database lock. The primary outcome—maximum pain score in the PACU—will be compared using Student’s *t*-test or the Wilcoxon rank-sum test. Secondary outcomes (e.g. opioid use, pain trajectories, QoR-15, length of stay, block duration) will be analysed using appropriate parametric or non-parametric tests. Repeated measures will be assessed via analysis of covariance (ancova), with baseline pain as a covariate. As postoperative pain variability between these procedures was smaller than interindividual variability in our preliminary study, they will be analysed together; however, exploratory subgroup analyses by surgical technique will be conducted if sample sizes allow. Missing data will not be imputed, but patterns and extent of missingness will be described. Sensitivity analyses may be performed if appropriate. Adverse events will be summarised by severity and relationship to the intervention.

### Monitoring

No formal data monitoring committee has been established for this trial, given the low-risk nature of the interventions and short-term follow-up. Trial oversight will be ensured by the sponsor through regular on-site and remote monitoring visits, as outlined in the monitoring plan. These visits will verify protocol compliance, data quality, and participant safety, following standard sponsor procedures and applicable regulatory requirements. No interim analyses or formal stopping rules are planned. Safety data will be continuously reviewed by the sponsor’s study team, who may suspend or terminate the trial if unexpected serious safety concerns arise.

### Ethics and dissemination

The trial was approved by the ‘Comité de Protection des Personnes Ouest IV’ ethics committee (approval reference: 25.01121.000344) in May 2025 and will be conducted in compliance with all relevant French and European regulatory requirements. Any substantial amendments to the protocol will be submitted for prior approval by the ethics committee (CPP) and communicated to investigators, regulatory authorities, and participants if necessary. Informed consent will be obtained before surgery by the investigator. Participants will be fully informed about the study objectives, procedures, potential risks and benefits, data protection measures, and their right to withdraw at any time. No additional biological specimens will be collected beyond standard care; therefore, no ancillary consent is required. Personal data will be collected using a secure, GDPR-compliant electronic system with pseudonymisation to maintain confidentiality. Access will be restricted to authorised study personnel. Confidentiality will be ensured throughout the study and during data archiving, which will comply with French regulations requiring a minimum 15-yr retention period. No specific post-trial care is planned beyond standard medical management. In the event of harm directly attributable to participation in the trial, compensation will be provided in accordance with French clinical trial insurance laws. Recruitment is scheduled to begin in September 2025 and to be completed by September 2026. At the time of manuscript submission, no patients have been enrolled. Results will be submitted to peer-reviewed journals and presented at national and international congresses. No professional writing assistance was used.

## Dissemination plans

Findings will be published in peer-reviewed journals and presented at national and international conferences to ensure dissemination to the wider clinical community.

## Protocol version

Protocol version 5.0, dated 25 March 2025.

## Roles and responsibilities

The sponsor manages the study’s administrative and regulatory requirements but has no role in its design, conduct, analysis, or reporting. The coordinating centre is the Department of Anaesthesia at Hôpital Privé Claude Galien, Quincy-sous-Sénart, France. The trial steering committee includes the principal investigator (Thomas Giral) and co-investigators from each participating centre. No endpoint adjudication committee or data safety monitoring board has been established, given the low-risk nature of the interventions and the use of routine, objective endpoints. Data management will be conducted locally at the coordinating site under the principal investigator’s supervision. Investigators are responsible for protocol adherence and data integrity at their own sites.

## Protocol, analysis plan and data availability

The full study protocol and the statistical analysis plan will be made available upon reasonable request to the corresponding author. De-identified individual participant data, including a data dictionary and statistical code, will be made available upon reasonable request to the corresponding author, after publication of the study results. Data will be shared in accordance with applicable data protection regulations.

## Authors’ contributions

Conceptualisation: TG, OM, JK

Writing – original draft, project administration: TG

Supervision, patient recruitment, data collection: all authors

Review and editing: OM, YE, DH, FC, KY, PD, AE, JK

Approved the final manuscript: all authors

## Funding

This work is supported by *GCS* *Ramsay Santé pour l’Enseignement et la Recherche*.

## Declarations of interest

The authors declare that they have no conflicts of interest.
